# Relationship of Klotho with cognition and dementia: Results from the NHANES 2011–2014 and Mendelian randomization study

**DOI:** 10.1038/s41398-023-02632-x

**Published:** 2023-11-01

**Authors:** Yue Wu, Shaoyuan Lei, Dongxiao Li, Zhongzhong Li, Yingzhen Zhang, Yansu Guo

**Affiliations:** 1 Beijing Geriatric Healthcare Center, Xuanwu Hospital, Capital Medical University, Beijing, China; 2 Department of Evidence-Based Medicine, Xuanwu Hospital, Capital Medical University, Beijing, China; 3 Department of Neurology, The Second Hospital of Hebei Medical University, Shijiazhuang, China; 4 Beijing Municipal Geriatric Medical Research Center, Beijing, China

**Keywords:** Diagnostic markers, Long-term memory

## Abstract

The relationships of Klotho levels with cognition and dementia are poorly understood. This study aimed to investigate the association between Klotho levels and cognitive function and to determine causality between Klotho and dementia using Mendelian randomization (MR). Based on data from the National Health and Nutrition Survey (NHANES) 2011–2014, this study consisted of 1875 older adults aged 60–79 years. Cognitive function was assessed by the digit symbol substitution test (DSST). We performed weighted multivariable-adjusted linear regression to assess the association between Klotho concentrations and cognitive function. Then, 2-sample MR was conducted to assess the causal relationship between Klotho and dementia. The inverse variance weighted (IVW) method was used as the primary analysis. We observed a positive association between serum Klotho concentrations and the results of the Digit Symbol Substitution test (DSST) (T2: β 2.16, 95% CI: 0.30–4.01, *P* = 0.03, T3: β 2.48, 95% CI: 0.38–4.57, *P* = 0.02) after adjusting for the covariates. Moreover, there was also a potential nonlinear relationship between Klotho and DSST. The IVW method showed that genetically predicted high Klotho levels were not significantly associate with any type of dementia, including Alzheimer’s disease (OR = 1.03, 95% CI: 0.96–1.10, *P* = 0.46), vascular dementia (OR = 1.04, 95% CI: 0.87–1.25, *P* = 0.66), frontotemporal dementia (OR = 0.73, 95% CI: 0.47–1.14, *P* = 0.16), or dementia with Lewy bodies (OR = 1.03, 95% CI: 0.87–1.23, *P* = 0.73). In the cross-sectional observational study, Klotho and cognitive function were significantly correlated; however, findings from MR studies did not indicate a causal relationship between Klotho and dementia.

## Introduction

As the population has aged, the incidence of dementia has increased. The prevalence of dementia is estimated to be as high as 7% in individuals over 65 years old [1]. Alzheimer’s disease (AD) is the most common form of dementia, accounting for approximately two-thirds of dementia cases. In addition to AD, other common types of dementia include vascular dementia (VD), frontotemporal dementia (FTD), and dementia with Lewy bodies (DLB) [2]. Cognitive function decline is manifested by a progressive deterioration of processing, attention, memory, and executive function, which is the hallmark feature of dementia [3]. Dementia and cognitive impairment place an immense burden not only on individuals but also on families, medical institutions and society. Klotho is encoded by the Klotho gene, which was originally described as possessing anti-aging capabilities [4]. There are two different forms of Klotho protein: membrane and secretory. The soluble form of Klotho protein (S-Klotho) is produced by proteolytic cleavage of Klotho transmembrane proteins. Compared to membrane Klotho protein, S-Klotho protein is more common in the human body and is found in urine, blood, and cerebrospinal fluid (CSF). Klotho plays an important role in calcium homeostasis as well as inhibiting insulin and insulin-like growth factor-1 signaling in cells [5, 6]. In addition to its association with aging, Klotho is also often related to changes in brain structure, declining cognitive function, and the occurrence of dementia.

Several epidemiological studies have assessed the relation of Klotho to cognitive function and dementia. However, there is still much uncertainty about the relationship. A longitudinal study showed that older adults with Alzheimer’s disease had significantly lower cerebrospinal fluid (CSF) Klotho concentrations than older adults with normal cognition [7]. A cross-sectional observational study on 94 subjects from Oregon Alzheimer’s Disease Center reported that the levels of Klotho protein in both serum and CSF were correlated with Mini-Mental State Examination (MMSE) scores [8]. In contrast, Gloria et al. reported that there was no association between Klotho levels and AD, but Klotho was strongly correlated with VD, especially in subjects with low levels of Klotho (less than 680 pg/mL) [9].

The existing observational studies have a few limitations, including small sample sizes, insufficient adjustment for some important variables, and lack of assessment of different types of dementia. Genetic Mendelian randomization (MR) analysis is a tool that uses genetic variants as instrumental variables to investigate the causality between clinical traits and disease phenotypes [10]. Because genetic alleles are randomly assigned during meiosis and are not influenced by environmental factors, MR is superior to observational studies in controlling for confounders and reverse causality [11]. MR studies are referred to as “nature-created randomized, double-blind trials,” which complement randomized controlled trials (RCTs). In view of inconsistent findings from observational studies and a lack of robust evidence from RCTs, MR studies may be a reliable supplementary method to explore the causal relationship between Klotho levels and dementia.

Therefore, we first used data from the National Health and Nutrition Examination Survey (NHANES) to investigate the association of serum Klotho concentrations with cognitive function after adjustment for several potential confounders. Then, we further evaluated the causal relationship between dementia and Klotho under the framework of the MR analysis.

## Methods

### Overall study design

The present study was composed of two parts. In the first part, we used the data from the National Health and Nutrition Examination Survey (NHANES) to investigate the association of Klotho levels with cognitive function after adjustment for several potential confounders. In the second part, we evaluated the causal effect of genetically predicted levels of Klotho on dementia using MR analysis of summary statistics from a genome-wide association study (GWAS).

#### Observational study

##### Sample population in NHANES

**Fig. 1:**
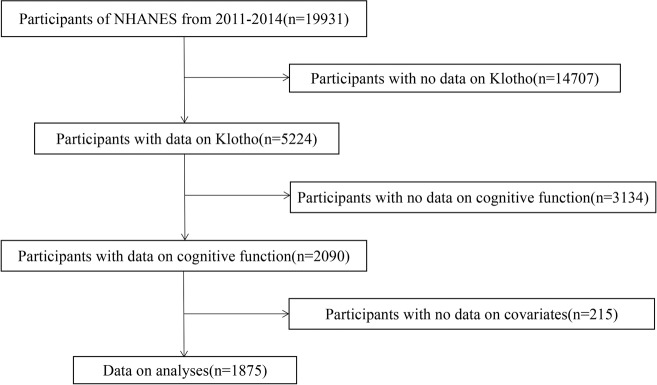
Flowchart of subject selection. The selection of eligible participants in the National Health and Nutrition Examination Survey (NHANES).

The data utilized in this study were from the National Health and Nutrition Examination Survey (NHANES) 2011–2014 dataset. NHANES is a national survey of health conducted by the Centers for Disease Control and Prevention (CDC) and the National Center for Health Statistics (NCHS) to analyze the health and nutrition of a nationally representative sample of noninstitutionalized US citizens [12]. Participants aged 60–79 years who had information on serum Klotho concentrations and underwent a complete cognitive function test were enrolled in the present study. After further excluding participants with missing information on covariates, 1875 subjects were included in the current study. The specific recruitment process is illustrated in Fig. 1. The NCHS Research Ethics Review Board approved the survey protocol, while all participants provided written informed consent.

#### Serum Klotho concentrations

Serum Klotho concentrations were the primary variable of interest. The samples were stored at −80 °C at the Centers for Disease Control and Prevention in Atlanta, GA. Between 2019 and 2020, they were shipped on dry ice to the Northwest Lipid Metabolism and Diabetes Research Laboratories at the University of Washington in Seattle, WA. Klotho quantification was based on an enzyme-linked immunosorbent assay kit (IBL International, Japan) [13]. Analysis results were automatically transmitted from the instrument to the Oracle management system in the laboratory, and the regional supervisor evaluated those results. Each sample was analyzed in duplicate, and the average of both values was calculated according to the manufacturer’s protocol. More than a 10% difference in duplicate values was flagged for remeasurement. The klotho detection limit for all samples was 6 pg/ml. There was no imputation for any samples since the final values were within this limit.

##### Cognitive function assessment

Cognitive function was assessed using the Digit Symbol Substitution test (DSST), which is an effective and validated measure for cognitive function [14]. To complete the DSST, study participants must correctly code a series of symbols in 120 seconds. The DSST was used to measure processing speed, sustained attention, and working memory, and it was a performance module from the Wechsler Adult Intelligence Scale (W AIS-III). It was scored between 0 and 105. In assessing the DSST results, the higher the score, the better the cognitive function.

##### Covariate information

Covariates included age (years), ethnicity (race), poverty income ratio (PIR), education level (school qualification), body mass index (BMI; kg/m^2^), physical activity (metabolic equivalent minutes per week of activity), smoking status, and alcohol status. All covariates were considered potential confounders in the relationship between serum Klotho concentrations and cognitive function. The participants were classified into two age groups: 60–69 and 70–79 years of age. The included racial groups were non-Hispanic White, non-Hispanic Black, Mexican American and other races (including other Hispanic and multiracial). Educational level was grouped as high school degree/equivalency or less, high school graduate/GED or equivalent, some college and college degree or more. NHANES participants self-reported their physical activity (PA) information using the Global Physical Activity Questionnaire (GPAQ), which is an effective PA surveillance instrument [15]. Participants without any PA and performing <600 MET min/week were classified as inactive [16]. Those performing ≥600 MET min/week were classified as active. It has been consistently demonstrated that PA levels of 600 MET min/week are associated with substantial health benefits [17]. The World Health Organization (WHO) classified BMI into four categories: underweight ( < 18.5 kg/m^2^), normal (18.5–24.9 kg/m^2^), overweight (25–29.9 kg/m^2^), and obese ( ≥ 30.0 kg/m^2^). There were four categories for the poverty-income ratio: <1 (below poverty level), 1–1.99, 2–3.99, and 4 (the richest). Alcohol consumption status was categorized into two strata: no and yes. Smoking status was categorized as never (reported smoking less than 100 cigarettes in lifetime), former (reported smoking more than 100 cigarettes in lifetime and not smoking at all now), and current (reported smoking less than 100 cigarettes in lifetime and currently smoking some days or every day).

##### Statistical analysis

According to NHANES analytic guidelines, sample weights, strata, and primary sampling units were used to account for the complex survey design. NHANES aimed to generate data that reflected the noninstitutionalized civilian population of the United States. For continuous variables, the study population characteristics are expressed as the mean (SE) or as a percentage. Participants were divided into tertiles according to their serum Klotho concentrations, with the lowest level representing the reference group (tertile 1, T1). The association between serum Klotho and cognitive function was examined using multiple linear regression analyses with the adjustment of all covariates. Restricted cubic splines were used to fit the shape of the dose–response association between serum Klotho levels and cognitive test scores. All statistical analyses were conducted using R software version 4.2.1. A two-sided *P* < 0.05 was considered to indicate statistical significance.

#### Mendelian randomization

##### Study design

**Fig. 2:**
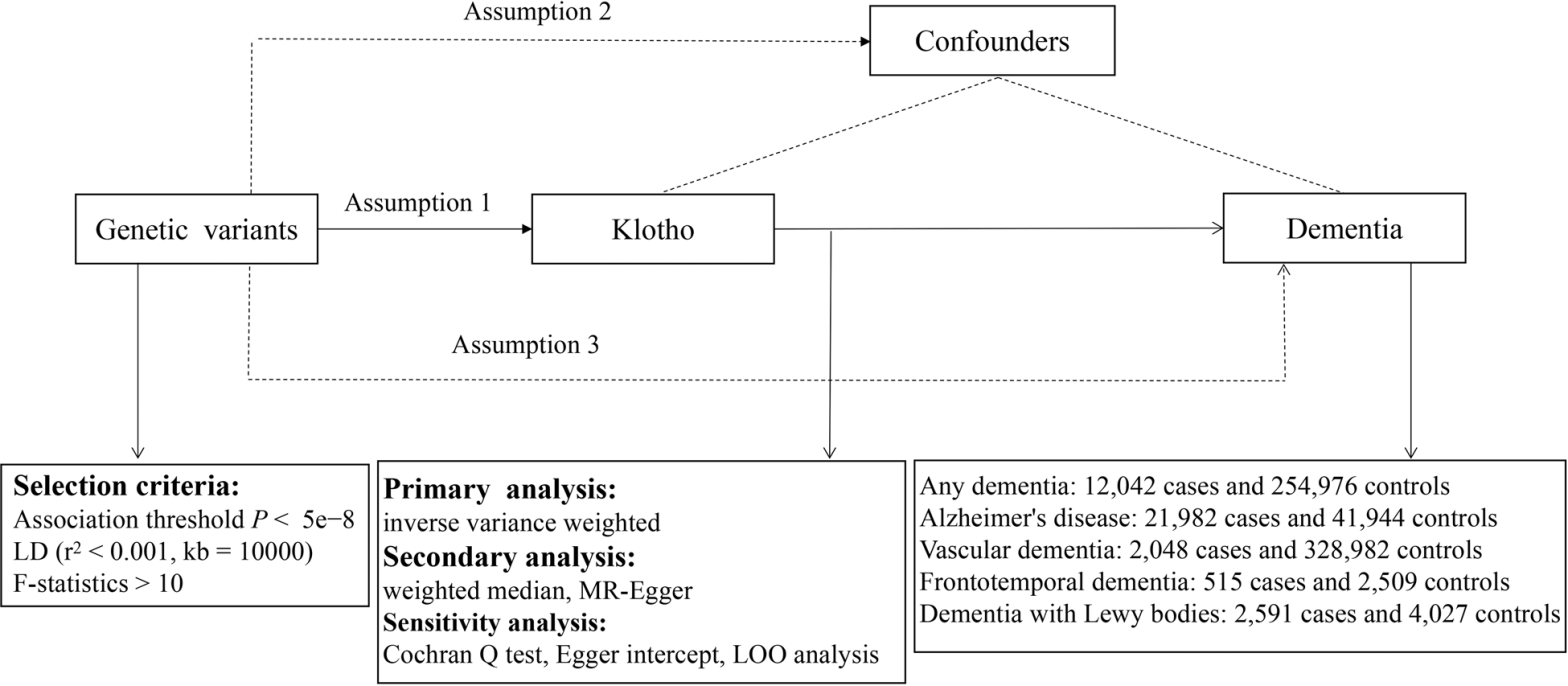
Principles of Mendelian randomization and assumptions. Assumption 1: exposure is robustly associated with genetic variants; Assumption 2: confounders are not associated with genetic variants; Assumption 3: genetic variants should influence the outcomes only mediated by the exposure of interest.

We performed a univariable two-sample MR analysis to determine the causal relationship between genetically predicted Klotho levels and dementia (i.e., any dementia, AD, VD, FTD, DLB) in our study. An overview of the research design is displayed in Fig. 2. MR analysis needs to satisfy three assumptions in order to investigate causal effects of exposure on the outcome: (1) the genetic variants were supposed to be correlated with Klotho levels; (2) they should not be associated with confounding factors; and (3) they should affect dementia only as mediated by the levels of Klotho. Furthermore, reverse-MR analysis was performed to avoid the possibility of reverse causality.

##### Genetic instrument selection

Genetic instruments for circulating Klotho were acquired from a meta-analysis of GWAS, which included data from 4376 European individuals. We selected genetic variants that preferentially satisfied three instrument assumptions of the MR analysis by the following procedures [18]. First, SNPs associated with circulating Klotho at a genome-wide significance level (*p* < 5e − 8) were extracted as instrumental variables (IVs). Second, SNPs in linkage disequilibrium were excluded (*r*^2^ threshold < 0.001 within a 10000 kb window), and the remaining SNPs were extracted from the outcome datasets. Third, we calculated F-statistics for each SNP to quantify the strength of the exposures and excluded weak ones (F-statistics < 10). Table S1 lists the SNPs used as IVs in this study.

##### Outcome data

A summary-level GWAS dataset including data for participants with any form of dementia was extracted from the Finn consortium, comprising 267,018 European-ancestry participants (12,042 patients and 254,976 control participants). GWAS summary statistics for AD were derived from the International Genomics of Alzheimer’s Project (IGAP), including 21,982 patients and 41,944 control participants [19]. From the Finn consortium, we obtained GWAS summary data for VD (2,048 patients and 328,982 control participants). FTD statistics were from an international multicenter study involving 515 patients and 2509 control participants [20]. GWAS data for DLB were obtained from another independent multicenter study with 2591 patients and 4027 control participants [21]. Table S2 summarizes the GWAS data in our study.

##### Statistical analysis

We utilized the inverse-variance weighted (IVW) method as the primary analysis, which provided the highest power but was not sensitive to horizontal pleiotropy. To correct for pleiotropy, the weighted median (WM) method and MR‒Egger regression were used as secondary methods. The odds ratios (ORs) and 95% confidence intervals (95% CIs) presented were scaled per standard deviation (SD) increase in circulating Klotho concentrations. To evaluate potential heterogeneity and horizontal pleiotropy, sensitivity analysis is essential. Cochran’s Q test was performed to evaluate the heterogeneity of effect sizes across genetic IVs. The MR‒Egger regression intercept was used to evaluate vertical pleiotropy [22]. We also performed leave-one-out (LOO) analyses to determine whether a single SNP influenced the MR estimate. To rule out reverse causality, we analyzed the influence of dementia on Klotho levels using reverse MR analysis.

## Results

### General characteristics of NHANES

Based on the NHANES sample weight, the analytic sample size in this study represented 37,005,278 American noninstitutionalized participants. According to the serum Klotho tertiles, the basic characteristics of the study subjects are summarized in Table 1. Sex, age, race, educational level, alcohol use, smoking status, physical activity, and poverty income ratio distribution were not significantly different among serum Klotho tertiles. In terms of DSST and BMI, significant differences were observed among the tertiles of Klotho.

**Table 1:** Survey-weighted characteristics of the overall NHANES sample according to serum Klotho tertiles (*N* = 1875).

Characteristic	Klotho Tertiles (pg/mL)				*P*
	Overall	Tertile 1 ≤ 704.2 ≤ 704.2	Tertile 2704.2–913.3	Tertile 3 >913.3	
DSST, mean scores (*SE)*	54.61 (0.63)	52.30 (0.85)	56.09 (0.83)	55.46 (1.07)	0.003
Age (%)					0.14
60-69	1202 (64.11)	373 (62.40)	413 (67.99)	416 (66.75)	
70-79	673 (35.89)	252 (37.60)	212 (32.01)	209 (33.25)	
Sex (%)					0.22
Male	905 (48.27)	319 (50.31)	308 (47.21)	278 (43.70)	
Female	970 (51.73)	306 (49.69)	317 (52.79)	347 (56.30)	
Race (%)					0.18
Non-Hispanic White	850 (45.33)	305 (81.01)	294 (81.81)	251 (76.97)	
Non-Hispanic Black	446 (23.79)	147 (7.23)	129 (6.42)	170 (9.55)	
Mexican American	175 (9.33)	51 (2.86)	64 (3.37)	60 (3.78)	
Other races	404 (21.55)	122 (8.89)	138 (8.40)	144 (9.69)	
Educational level (%)					0.12
College degree or more	436 (23.25)	130 (30.14)	144 (30.72)	162 (33.54)	
High school degree/equivalency or less	442 (23.57)	161 (17.12)	139 (11.98)	142 (14.37)	
High school graduate/GED or equivalent	429 (22.88)	151 (23.08)	135 (18.54)	143 (20.55)	
Some college	568 (30.29)	183 (29.65)	207 (38.75)	178 (31.54)	
Smoking status (%)					0.44
Current	261 (13.92)	94 (13.26)	88 (10.12)	79 (13.53)	
Former	713 (38.03)	253 (40.90)	246 (40.39)	214 (37.64)	
Never	901 (48.05)	278 (45.84)	291 (49.49)	332 (48.83)	
Alcohol status (%)					0.98
No	778 (41.49)	254 (33.74)	261 (33.23)	263 (33.09)	
Yes	1097 (58.51)	371 (66.26)	364 (66.77)	362 (66.91)	
Physical activity (%)					0.65
Active	970 (51.73)	305 (53.65)	340 (57.62)	325 (56.33)	
Inactive	905 (48.27)	320 (46.35)	285 (42.38)	300 (43.67)	
Body mass index (%)					0.04
Normal	447 (23.84)	132 (19.26)	149 (21.69)	166 (27.88)	
Obese	747 (39.84)	244 (36.85)	256 (42.89)	247 (37.53)	
Overweigh	662 (35.31)	241 (42.99)	212 (34.03)	209 (33.87)	
Underweight	19 (1.01)	8 (0.90)	8 (1.38)	3 (0.72)	
Poor income ratio (%)					0.32
<1	317 (16.91)	107 (8.07)	103 (7.80)	107 (10.24)	
1–1.99	524 (27.95)	178 (21.03)	171 (19.96)	175 (21.78)	
2–3.99	509 (27.15)	169 (28.37)	184 (32.74)	156 (24.41)	
≥4	525 (28)	171 (42.53)	167 (39.50)	187 (43.57)	

### Association between serum Klotho concentrations and cognitive function

As shown in Table 2, multivariate linear regression models were used to evaluate the association between serum Klotho concentrations and cognitive function. According to Model 2, the higher the Klotho concentration was, the better the DSST performance (T2: β 2.98, 95% CI: 1.24–4.72, *P* = 0.002, T3: β 3.02, 95% CI: 0.79–5.26, *P* = 0.01). After adjusting for all covariates, there was still a positive association between Klotho and DSST performance (T2: β 2.16, 95% CI: 0.30–4.01, *P* = 0.03, T3: β 2.48, 95% CI: 0.38–4.57, *P* = 0.02, *P*- trend = 0.022).

**Table 2:** Multiple linear regression analysis on the association between serum Klotho and DSST.

	serum Klotho (pg/mL)			*P*-trend
Characteristic	Tertile 1 ≤ 704.2	Tertile 2704.2−913.3	Tertile 3 > 913.3	
Model 1	1.00	3.79 (1.68, 5.90) ***	3.16 (0.62, 5.71) *	0.013
Model 2	1.00	2.98 (1.24, 4.72) **	3.02 (0.79, 5.26) *	0.009
Model 3	1.00	2.16 (0.30, 4.01) *	2.48 (0.38, 4.57) *	0.022

*P < 0.05, **P < 0.01, ***P < 0.001.

Model 1: unadjusted model.

Model 2: adjusted for age, sex, and race.

Model 3: further adjusted for BMI, alcohol status, smoking status, educational level, family income to poverty ratio, and physical activity.

### Dose‒response relationship analysis

**Fig. 3:**
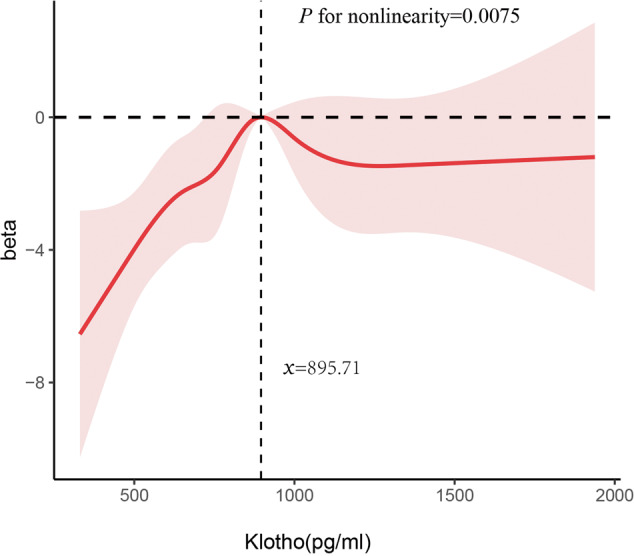
Restricted cubic spline plot of the association between serum Klotho levels and DSST scores. The associations were adjusted for age, sex, race, educational level, smoking status, alcohol status, physical activity, body mass index, and poor income ratio.

Restricted cubic splines (RCSs) were used to analyze the relationship between serum Klotho concentrations and DSST scores. Based on this relationship, a nonlinear model provided a better explanation of the relationship than a linear model after adjusting for all covariates (*P* = 0.0075) (Fig. 3). The DSST scores gradually increased on the left side of the inflection point (895.71 pg/mL), while on the right side, they marginally decreased and reached saturation.

### MR of Klotho and dementia

**Fig. 4:**
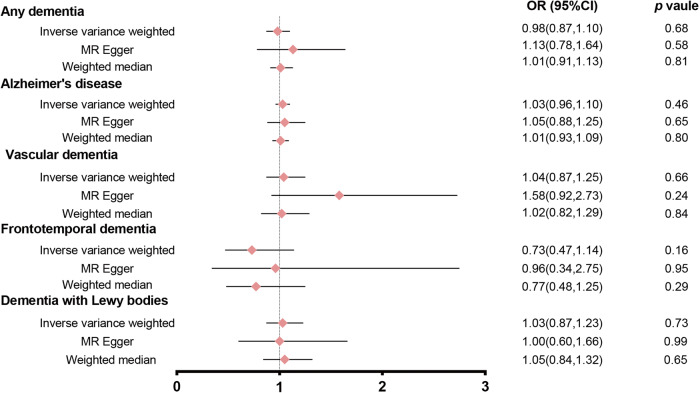
Forest plot of the MR study investigating the effect of Klotho levels on any type of dementia. There was no significant causal association between the levels of Klotho and any type of dementia.

In the first step, we pooled effect estimations from individual genetic instruments based on the IVW method. Under the IVW model, there was no significant causal association between the levels of Klotho and AD risk (OR = 1.03, 95% CI: 0.96–1.10, *P* = 0.46). Similar findings were found for the risk of Klotho associated with any dementia, VD, FTD and DLB. In various kinds of dementia, the findings of MR-Egger and MW were consistent with the IVW method (Fig. 4). As a result of the sensitivity analysis, no horizontal pleiotropy (*P* intercept > 0.05) or heterogeneity (*P* Q > 0.05) was discovered among the selected instruments (Table S3). These results were confirmed by LOO analysis (Fig. S1). Only one independent SNP was identified for VD and FTD, so we did not conduct the reverse MR analysis in VD and FTD. There were no reverse associations of genetic liability to other forms of dementia with Klotho concentrations (Table S4, Fig. S2).

## Discussion

To the best of our knowledge, this study is the first comprehensive investigation of the relationship between cognitive function and dementia risk with Klotho concentrations based on large-scale observational research data and MR analysis of large-scale genetic data. We examined the association between Klotho concentrations and cognitive function. Furthermore, we investigated the potential causal relationship between Klotho levels and dementia. The results revealed that serum Klotho had a positive correlation with DSST scores. Studies in a range of clinical populations have shown that DSST is sensitive to detect both cognitive dysfunction and changes in cognitive dysfunction [23], especially in cognitive domains of attention, psychomotor speed and visuospatial abilities. Surprisingly, we found a potential nonlinear relationship between Klotho levels and DSST scores. However, the MR analysis results did not support a causal association between Klotho concentrations and dementia risk. More specifically, our results indicate that increases in Klotho concentrations were not associated with protection against dementia.

Studies on the association between Klotho concentrations and cognitive function with large-scale, representative populations are scarce. At present, only two clinical studies have included a large sample size of over 100 individuals to evaluate the relationship between Klotho concentrations and cognitive function in older adults, and the results are inconsistent. One study from the CHIANTI study enrolled 833 participants aged 55 or older and reported that the concentrations of Klotho were not significantly cross-sectionally associated with the Trail-Making Test A (Trails A) and Trail-Making Test B (Trails B), which measured cognitive domains, including working memory, sustained attention and psychomotor performance [24]. Another study reported by Sanz et al. showed that participants aged 70 or older with lower Klotho concentrations showed a trend toward worse processing speed (*r* = 0.256) [25], which is consistent with our observational results. In this study, we innovatively analyzed the dose‒response relationship between serum Klotho and cognitive function. Before the inflection point, the DSST scores gradually improved along with the elevation of Klotho concentrations. However, after the inflection point, DSST scores marginally decreased and reached saturation. In accordance, higher serum Klotho concentrations do not necessarily mean better cognitive performance. To the best of our knowledge, few studies have previously reported this nonlinear relationship. This nonlinear relationship may explain the inconsistent correlation between Klotho and cognition reported in previous observational studies.

However, across all MR methods, we did not find evidence of a causal relationship between Klotho levels and dementia. To ensure the validity of MR studies, horizontal pleiotropy and heterogeneity should be adequately checked. Our analysis of horizontal pleiotropy was conducted by performing the MR‒Egger intercept test, which indicated that there was no horizontal pleiotropic effect in dementia. Cochrane’s Q test and leave-one-out sensitivity analyses confirmed the robustness of the significant findings. Moreover, we used bidirectional MR analysis to eliminate the possibility of reverse causation. Concerning the discrepancies between our observational and MR results, using only one inaccurate measure of obesity and physical activity may only have moderately reduced the confounding effects on the outcome [26]. Significantly, MR analysis provided deeper evidence. Consequently, no causal relationships between Klotho levels and dementia can be inferred.

There are several strengths of the present study, including its large sample size, the inclusion of several types of dementia, and the use of the above SNPs as genetic instrumental variables. Importantly, MR analysis enabled the evaluation of the causal relationship between Klotho and dementia. Compared with observational studies, MR analysis significantly reduces biases such as reverse causation and confounding. However, some limitations of this study warrant mentioning. First, although no directional pleiotropy was detected in our study, there might still exist some potential pleiotropies. This is a typical issue in MR research and a source of bias. Second, the MR analysis estimates exposure effects over a lifetime, not at a specific moment in time. It is unclear whether an increase in Klotho levels can prevent dementia at a specific time in life. Third, our observational study showed that there was a nonlinear relationship between Klotho and cognitive function. The MR study, however, examined only linear causal associations, so nonlinear causality cannot be ruled out. Fourth, our MR study was conducted on individuals of European descent, while the cross-sectional study was conducted on a multiethnic US population. To eliminate potential confounding factors for population heterogeneity, a study of the same ethnicity is needed in the future. Fifth, our study is essentially a statistical analysis and lacks certain components of wet experiments using blood samples from dementia patients.

## Conclusion

Our MR analysis did not show a genetic cause-and-effect relationship between Klotho and dementia in individuals of European descent, even though an observational study suggested a significant association between serum Klotho and cognitive function in a multiethnic US population. The observational results may be subject to bias, including uncontrolled confounding factors. As a result, based on the present findings, there is no indication that the increase in Klotho levels may be beneficial for the prevention of dementia.

### Supplementary information

supplementary tables and figures

## Data Availability

All data analyzed in this study can be obtained by a reasonable request to the corresponding author.

## Code availability

Codes used in current study are available from the corresponding author on reasonable request.
