# Supplementary material for: Relationship of Klotho with cognition and dementia: Results from the NHANES 2011–2014 and Mendelian randomization study
Source: Transl Psychiatry. 2023 Nov 1;13:337. doi: 10.1038/s41398-023-02632-x (PMC10620156; doi:10.1038/s41398-023-02632-x)
Supplement: Supplementary file 1 — supplementary tables and figures [file 41398_2023_2632_MOESM1_ESM.docx]

**Supplementary files**

**Table S1.** Characteristics of selected SNPs for circulating Klotho.

| SNPs | Effect allele | Other alleles | R^2^ (%) | F | Beta | SE | *P* value |
| --- | --- | --- | --- | --- | --- | --- | --- |
| rs12607664 | T | G | 2.54 | 114.1 | 0.24 | 0.022 | 2.28e-27 |
| rs8176672 | T | C | 2.20 | 98.3 | 0.41 | 0.041 | 2.11e-23 |
| rs532436 | G | A | 1.41 | 62.3 | 0.20 | 0.026 | 5.86e-15 |
| rs1056008 | C | T | 1.32 | 58.6 | 0.18 | 0.024 | 1.80e-14 |
| rs7333961 | A | G | 0.94 | 41.6 | -0.33 | 0.051 | 1.73e-10 |
| rs881301 | C | T | 0.68 | 30.0 | -0.12 | 0.021 | 2.23e-08 |

**Table S2.** The GWAS details of dementia in the Mendelian randomization.

| Trait | Population | Cases | Controls | Sample size | PubMed ID or web source |
| --- | --- | --- | --- | --- | --- |
| Any dementia | European | 12,042 | 254,976 | 267,018 | www.finngen.fi/en |
| Alzheimer's disease | European | 21,982 | 41,944 | 63,926 | DOI: 10.1038/s41588-019-0358-2 |
| Vascular dementia | European | 2,048 | 328,982 | 331,030 | www.finngen.fi/en |
| Frontotemporal dementia | European | 515 | 2,509 | 3,024 | DOI: 10.1038/ng.536 |
| Dementia with Lewy bodies | European | 2,591 | 4,027 | 6,618 | DOI: 10.1038/s41588-021-00785-3 |

**Table S3.** Heterogeneity and pleiotropy tests for the Klotho levels with dementia.

| **Outcome** | **Cochrane’s *Q* test** | | | **MR-Egger intercept test** | |
| --- | --- | --- | --- | --- | --- |
|  | **Q-value** | ***P* _Q_** |  | **Intercept** | ***P* _intercept_** |
| Any dementia | 6.196 | 0.102 |  | -0.032 | 0.492 |
| Alzheimer's disease | 2.264 | 0.812 |  | -0.005 | 0.822 |
| Vascular dementia | 2.808 | 0.422 |  | -0.091 | 0.251 |
| Frontotemporal dementia | 0.363 | 0.834 |  | -0.065 | 0.675 |
| Dementia with Lewy bodies | 4.698 | 0.454 |  | 0.008 | 0.894 |

**Table S4.** Reverse causal relationship of circulating Klotho with dementia performed by MR.

| **Exposure** |  | **Method** | **β (95%CI)** | ***p* value** |  | ***P* _Q_** |  | ***P* _intercept_** |
| --- | --- | --- | --- | --- | --- | --- | --- | --- |
| Any dementia |  | IVW | -0.16 (-0.35, 0.04) | 0.11 |  | 0.28 |  |  |
|  |  | MR Egger | -0.74 (-1.32, -0.16) | 0.07 |  |  |  | 0.11 |
|  |  | WM | -0.18 (-0.41, 0.05) | 0.13 |  |  |  |  |
| Alzheimer's disease |  | IVW | -0.05 (-0.17, 0.07) | 0.42 |  | 0.59 |  |  |
|  |  | MR Egger | -0.06 (-0.60, 0.47) | 0.82 |  |  |  | 0.95 |
|  |  | WM | 0.03 (-0.13, 0.19) | 0.68 |  |  |  |  |
| Dementia with Lewy bodies |  | IVW | 0.03 (-0.07, 0.12) | 0.59 |  | 0.81 |  |  |
|  |  | MR Egger | 0.20 (-1.07, 1.46) | 0.79 |  |  |  | 0.81 |
|  |  | WM | 0.03 (-0.08, 0.14) | 0.56 |  |  |  |  |

**Figure S1.** Leave-one-out analysis for Klotho on any dementia (A), AD (B), VD (C), FTD (D), and DLB (E).


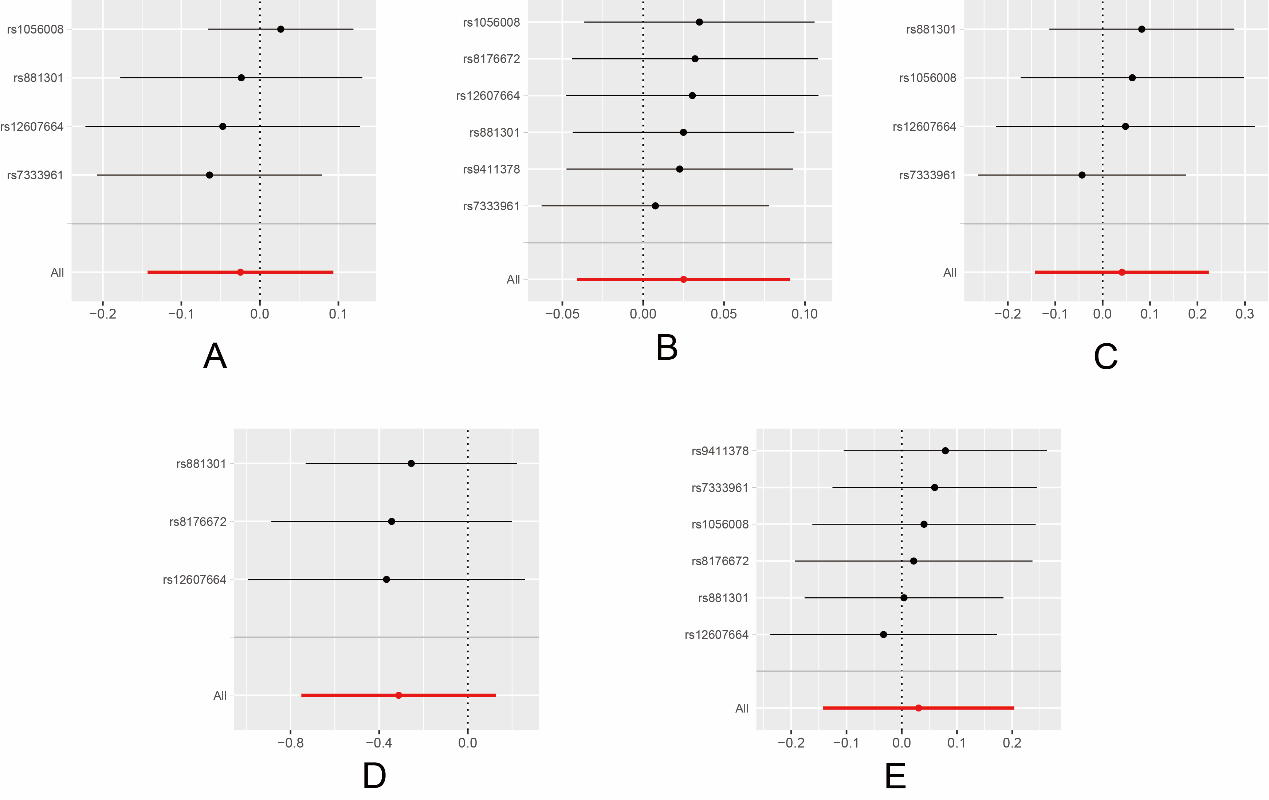


**Figure S2.** Reverse leave-one-out analysis for Klotho on any dementia (A), AD (B), and DLB (C).

**
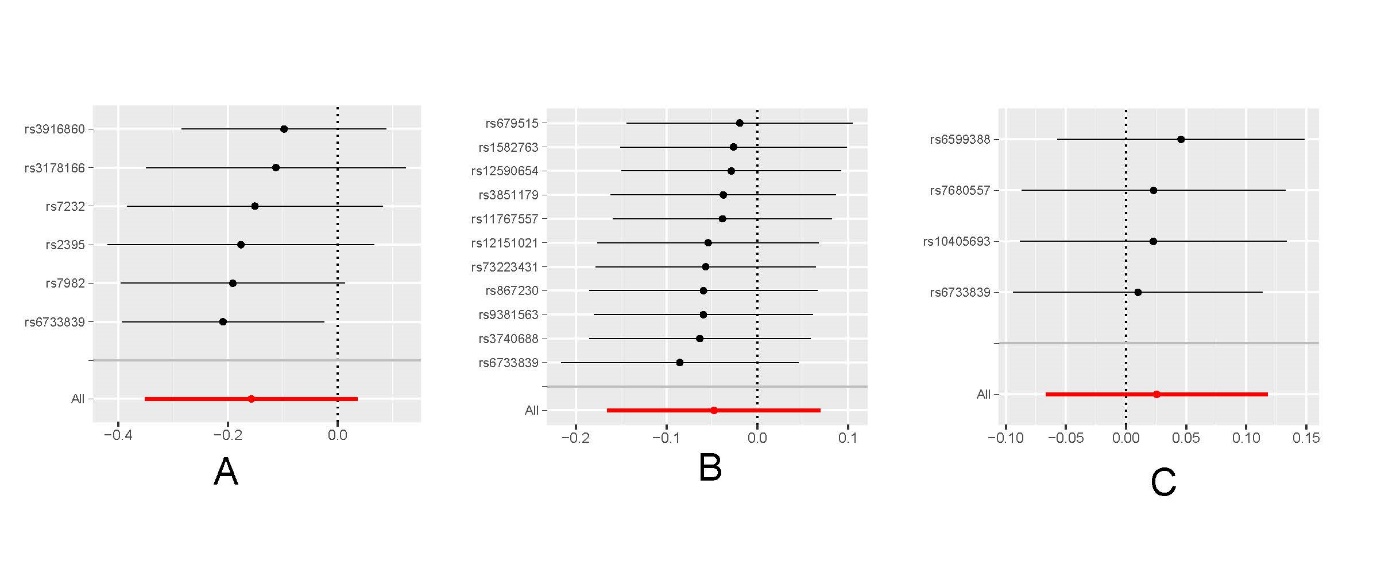
**
